# Application of Feature Definition and Quantification in Biological Sequence Analysis

**DOI:** 10.2174/1389202924666230816150732

**Published:** 2023-10-27

**Authors:** Weiyang Chen, Weiwei Li

**Affiliations:** 1 School of Cyber Science and Engineering, Qufu Normal University, Qufu, China;; 2 Qilu Institute of Technology, Shandong, China

**Keywords:** Biological sequence, sequence alignment, alignment-free analysis, texture feature, feature definition, quantitative research

## Abstract

Biological sequence analysis is the most fundamental work in bioinformatics. Many research methods have been developed in the development of biological sequence analysis. These methods include sequence alignment-based methods and alignment-free methods. In addition, there are also some sequence analysis methods based on the feature definition and quantification of the sequence itself. This editorial introduces the methods of biological sequence analysis and explores the significance of defining features and quantitative research of biological sequences.

## 1 INTRODUCTION

Biological sequences usually refer to nucleotides or amino acid sequences. A widely recognized rule is that sequence determines structure, and structure determines function. Therefore, it is widely recognized that biological sequences are the most fundamental data. Biological sequence analysis is one of the most fundamental and important tasks in bioinformatics [1]. We can study the structure, function, and evolutionary relationships among species within biological sequences by analyzing biological sequences. In biological sequence analysis, the traditional method is to study the similarity between sequences through sequence alignment. By analyzing the similarity among different sequences, the structure and function of sequences with unknown structures or functions can be predicted. The method of sequence alignment usually requires defining a scoring algorithm and inserting spaces into the sequence to obtain the maximum score between sequences during the alignment process. Unlike sequence alignment, there is another type of analysis method that is the alignment-free biological sequence comparison method [2].

Alignment-free biological sequence comparison methods do not require defining scoring algorithms or inserting spaces. Alignment-free biological sequence comparison methods generally obtain the similarity between different sequences through some statistical analysis [3], probabilistic methods [4, 5] or mutual information calculation [6]. The methods based on sequence alignment and alignment-free have their own advantages and disadvantages and are respectively applied to meet different needs [7].

When analyzing sequences through alignment-free methods, the digital encoding of biological sequences facilitates logical and numerical operations between sequences. The methods of digital encoding include numerical encoding [8], binary encoding [9], and so on. Digital encoding is essential for analyzing features of biological sequences. After digitizing and encoding biological sequences, potential features in biological sequences can also be analyzed by studying the patterns and frequency of occurrence between numerical values [10]. For example, texture features can be computed from digital sequence [11]. Texture features are a very important type of feature in image processing [12, 13]. They are used to analyze and describe the differences in structure and texture exhibited by different images. Applying the method of calculating texture features on images to biological sequences can yield some advantages [10]. Firstly, various texture features can be quantified for each biological sequence. Secondly, these texture features quantified from each biological sequence can also be used for similarity comparison among different sequences and the construction of phylogenetic trees. In addition, texture features can also contain other information. For example, the frequency of 2-mers, which is a short sequence with a length of 2, can be easily calculated in texture features. The co-occurrence matrix is used when calculating texture features. It represents the frequency at which two characters at a certain distance appear simultaneously. The calculation result obtained by setting the distance parameter to 1 is the frequency of 2-mers. Moreover, the method of defining texture features is more flexible, making it easy to calculate the frequency of the simultaneous occurrence of bases at certain distances.

For the definition and quantification of texture features in biological sequences, it is possible to explore the potential characteristics of each sequence and provide quantitative values. Although the current research on texture features of biological sequences is still in the exploratory stage. But the research on these texture features in images is already very mature. For images, each texture feature reflects some of the visual effects and meanings inherent in the image itself. For example, the contrast texture feature of an image represents the difference in color and brightness among different regions in an image, and the greater the difference, the higher the contrast of the image. An image with high contrast gives the impression of being more dazzling, bright, and the objects in the image are more prominent. The research on these texture features in images is already very mature. In the future, it will be very meaningful to conduct biological research on the quantified features from biological sequences. These features, as well as the definitions of the mathematical formulas behind them, reflect the arrangement and combination characteristics of numbers in a sequence. Whether they are related to the deep biological characteristics behind the sequence is highly worthy of our in-depth research. Characterizing and quantifying the biological characteristics and meanings of biological sequences through mathematical formulas will help us gain a deeper understanding of the mysteries of biological sequences. Just as the texture features of an image reflect its visual effect, it is worth studying whether the texture features of biological sequences reflect certain effects or characteristics.
